# Competing‐risks model for predicting the prognosis of penile cancer based on the SEER database

**DOI:** 10.1002/cam4.2649

**Published:** 2019-10-27

**Authors:** Jin Yang, Zhenyu Pan, Yujing He, Fanfan Zhao, Xiaojie Feng, Qingqing Liu, Jun Lyu

**Affiliations:** ^1^ Clinical Research Center The First Affiliated Hospital of Xi'an Jiaotong University Xi'an Shaanxi China; ^2^ School of Public Health Xi'an Jiaotong University Health Science Center Xi'an Shaanxi China; ^3^ Department of Pharmacy The Affiliated Children Hospital of Xi'an Jiaotong University Xi'an Shaanxi China; ^4^ Department of Thoracic Surgery Nanfang Hospital Southern Medical University Guangzhou Guangdong China

**Keywords:** competing‐risks model, fine‐gary, penile cancer, prognostic factors, SEER database

## Abstract

**Objectives:**

This study performed a competing‐risks analysis using data from the SEER database on penile cancer patients with the aim of identifying more accurate prognostic factors.

**Methods:**

Data on patients with penile cancer were extracted from the SEER database. A univariate analysis used the cumulative incidence function and Gray's test, while multivariate analysis was performed using the Fine‐Gray model. Cumulative hazards were compared with a competing‐risks model constructed using Kaplan‐Meier estimation.

**Results:**

The multivariate Fine‐Gray analysis indicated that being black (HR = 1.51, 95%CI: 1.10‐2.07, *P* = .01), AJCC stage II (HR = 1.94, 95%CI: 1.36‐2.77, *P* < .001), AJCC stage III (HR = 1.98, 95%CI: 1.34‐2.91, *P* < .001), tumor size > 5 cm (HR = 2.23, 95%CI: 1.33‐3.72, *P* < .05), and TNM stages N1 (HR = 2.49, 95%CI: 1.71‐3.61, *P* < .001), N2 (HR = 3.25, 95%CI: 2.18‐4.84, *P* < .001), N3 (HR = 5.05, 95%CI: 2.69‐9.50, *P* < .001), and M1 (HR = 2.21, 95%CI: 1.28‐3.84, *P* < .05) were statistically significant. The results obtained using multivariate Cox regression were different, while Kaplan‐Meier curve analysis led to an overestimation of the cumulative risk of the patient.

**Conclusions:**

This study established a competing‐risks analysis model for the first time based on the SEER database for the risk assessment of penile cancer patients. The results may help clinicians to better understand penile cancer and provide these patients with more appropriate support.

## Graphical abstract

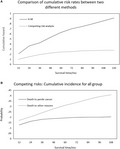

## 1 INTRODUCTION

Cancers of the male genital system account for nearly one‐third of all cancers in males, including prostate, testicular, and penile cancers.^1^ Penile cancer is a malignant tumor that is rare in developed countries, especially in the United States and in European countries.^2^, ^3^, ^4^ The morbidity and mortality rates of penile cancer have been low and stable in Nordic countries.^3^ There were 2030 new cases of penile cancer in the United States in 2016, with 340 deaths.^5^ In contrast, penile cancer accounts for 10%‐20% of malignant tumors in males in Africa, Asia, and South America.^6^

Squamous cell carcinoma is the most common malignant lesion of the penis.^7^, ^8^, ^9^ Although there have been some reports of risk factors associated with penile cancer survival, most of them were based on Cox proportional‐hazards regression models and Kaplan‐Meier estimates.^10^, ^11^ Performing a follow‐up or making observations of a two‐state model from a start event to an event of interest is a common design and analysis strategy, and Kaplan‐Meier estimates, log‐rank tests, and Cox regression are widely used for such single events of interest**.** However, those statistical analyses involve only one type of event. In medical research, the observed endpoints are rarely single, but there are multiple endpoints. The occurrence of competing events “blocks” the occurrence of the ending event of interest and forms a “competing relationship.” For example, in cardiovascular disease mortality studies, patients may die from cardiovascular disease or die from other causes such as cancer, suicide, etc The frequency of comorbidities may be especially high in older people; for example, the risk of death from heart disease and cerebrovascular disease increases with age in patients with nonsmall‐cell lung cancer.^12^ Traditional survival analysis will treat such competing risks by censoring, which will lead to miscalculations of the survival function.^13^ This is because the Kaplan‐Meier method and the Cox method treat other competing events as censored, and there may be conclusions that are estimated to be high or even contrary with the facts, also called competitive risk bias.^14^ These considerations indicate the need to use a competing‐risks model to handle multiple endpoints.

This study conducted a competing‐risks analysis using data from the SEER database on penile cancer patients with the aim of identifying more accurate prognostic factors.

## 2 METHODS AND MATERIALS

### 2.1 Patients

Data on patients with penile cancer were extracted from the SEER database using version 8.3.5 of the SEER*Stat software. The SEER program includes 18 registries that cover 30% of the United States population and collects demographic, clinical, and outcome information on all cancers diagnosed in representative geographic regions and subpopulations of the United States.^15^, ^16^ We queried the SEER program database for records from 2004 to 2015 using inclusion criteria of an age at diagnosis ≥ 18 years and the following site codes of the International Classification of Diseases for Oncology, Third Edition (ICD‐O‐3): C60.1 (glans penis), C60.2 (body of penis), C60.8 (overlapping lesion of penis), and C60.9 (penis NOS). The exclusion criteria were (a) no surgery, diagnosis, or microscopy confirmation, (b) only autopsy findings, or (c) incomplete variables.

The following information was collected for each patient: age at diagnosis, race, marital status, AJCC stage, surgery status, radiation status, tumor size, N stage, M stage, examination status of regional lymph nodes, primary site, survival time, and cause of death. We adopted the sixth edition of the AJCC staging system since this was used for recording data in the SEER database from 2004.

### 2.2 Statistical analysis

Continuous data are presented as the mean ± standard‐deviation values, and categorical data are presented as frequencies and proportions. We regarded other causes of death as competing events in our analysis of competing risks. When there is a competiting risk, the outcome is not only survival, death. Cumulative incidence function, CIF_k_(*t*) = Pr(*T* ≤ *t*, *D* = *k*), represents the probability of the k event before time t and other types of events.^17^ The comparison between the cumulative incidences of the groups is checked by the Gray test.^17^ Univariate analysis was performed using the cumulative incidence function (CIF) to show the probability of each event and Gray's test to estimate the difference in the CIF between groups.^18^ Multivariate analysis with the Fine‐Gray model was used to identify factors affecting the cumulative incidence of penile cancer. The Fine‐Gray model is designed to fit the cumulative incidence of events of interest.^19^ It is suitable for personal risk prediction research, tends to estimate disease risk and prognosis, and is suitable for establishing clinical prediction models and risk scores.^20^ We also compared the results from a Cox regression model with those from the Fine‐Gray model. The cumulative hazard was compared with a competing‐risks model constructed using Kaplan‐Meier estimation. Finally, according to the Bayesian information standard, we repeated the multivariate analysis using age as a time‐varying covariate.

All statistical analyses were performed using SPSS (version 24.0, SPSS), Empower Stats (version 2018‐12‐22; http://www.empowerstats.com/cn/), and R statistical software (version 3.5.0; https://www.r-project.org/). The “cmprsk” R package was used to construct the model. All statistical tests were two‐sided, with *P* < .05 considered to be indicative of statistical significance.

The SEER database can be accessed free of charge, and this study was exempted from obtaining informed consent from the included patients by the institutional research committee of the First Affiliated Hospital of Xi'an Jiaotong University.

## 3 RESULTS

### 3.1 Patient characteristics

**Figure 1:**
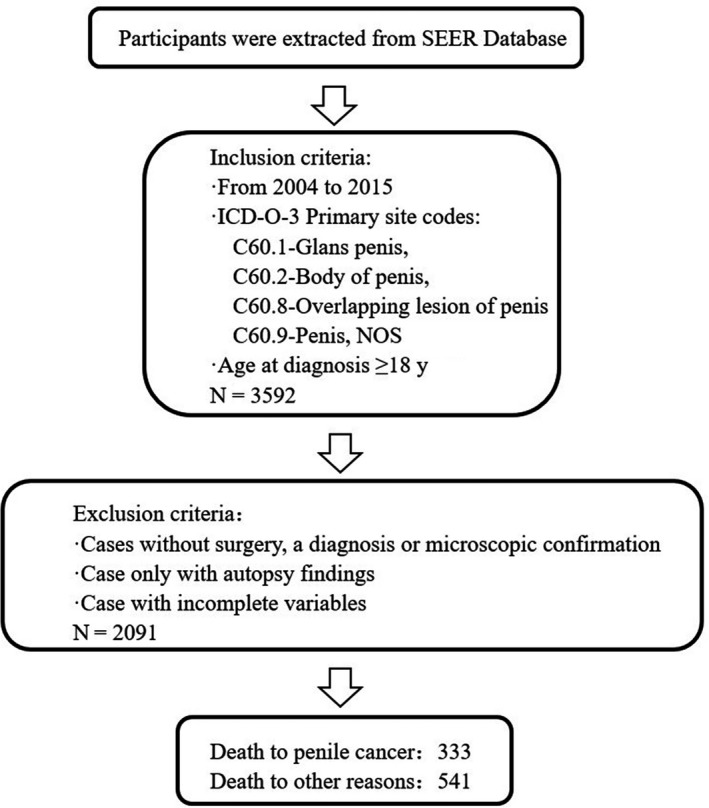
Flow diagram for the selection of patients

Of the 2091 eligible patients, 541 died of other causes such as other cancers, suicide, and accidents, accounting for 25.87% of the total. Death due to other reasons was considered a competing event; 333 died of penile cancer, accounting for 15.93% of the total. Those who died of penile cancer were aged 66.00 ± 13.85 years. Most of the patients were married (n = 191, 15.4%), were AJCC stage III (n = 97, 22.7%), had received surgery (n = 314, 15.6%), had not received radiation or had an unknown radiation status (n = 280, 14.7%), had a tumor size of >3 cm and ≤5 cm (n = 118, 19.1%), were TNM stage N0 (n = 171, 10.3%), were TNM stage M0 (n = 302, 14.9%), had not had their regional lymph nodes examined (n = 193, 12.7%) and had an ICD‐O‐3 code of C60.9 (penis NOS) at the primary site (n = 158, 16.0%). The median follow‐up time was 27 months. The results are provided in detail in Table 1 and Figure 1.

**Table 1:** Patients characteristics and demographics

Variables	ALL	Death to penile cancer	Death to other reasons
Age	67 ± 14.24	66 ± 13.85	75 ± 11.79
Race			
White	1772	274 (15.5)	450 (25.4)
Black	201	44 (21.9)	53 (26.4)
Other	118	15 (12.7)	38 (32.2)
Marital status			
Married	1242	191 (15.4)	299 (24.1)
Unmarried	381	64 (16.8)	76 (19.9)
DSW	468	78 (16.7)	166 (35.5)
AJCC stage			
I	922	64 (6.9)	237 (25.7)
II	543	89 (16.4)	147 (27.1)
III	428	97 (22.7)	110 (25.7)
IV	198	83 (41.9)	47 (23.7)
Surgery			
Yes	2012	314 (15.6)	515 (25.6)
No/Unknown	79	19 (24.1)	26 (32.9)
Radiation			
Yes	182	53 (29.1)	43 (23.6)
No/Unknown	1909	280 (14.7)	498 (26.1)
Tumor size(cm)			
≤1	237	18 (7.6)	52 (21.9)
1 < T≤ 3	866	103 (11.9)	224 (25.9)
3 < T≤5	617	118 (19.1)	171 (17.7)
>5	371	94 (25.3)	94 (25.3)
N			
N0	1661	171 (10.3)	448 (27.0)
N1	144	43 (29.9)	36 (25.0)
N2	159	59 (37.1)	30 (18.9)
N3	127	60 (47.2)	27 (21.3)
M			
M0	2025	302 (14.9)	525 (25.9)
M1	66	31 (47.0)	16 (24.2)
Regional nodes examined			
Yes	573	140 (24.4)	97 (16.9)
No	1518	193 (12.7)	444 (29.2)
Primary site			
Glans penis	861	136 (15.8)	216 (25.1)
Body of penis	134	19 (14.2)	38 (28.4)
Overlapping lesion of penis	108	20 (18.5)	30 (27.8)
Penis NOS	988	158 (16.0)	257 (26.0)

Abbreviations: AJCC, American Joint Committee on Cancer; DSW, divorced & separated &widowed; NOS, not otherwise specified.

### 3.2 Univariate analysis of the prognosis of penile cancer

The univariate analysis included Gray's test and the CIF. When competing risks were present, the results of Gray's test showed that age, race, AJCC stage, surgery status, radiation status, tumor size, N stage, M stage, and the examination status of regional lymph nodes exerted statistically significant effects on penile cancer (*P* < .05). The CIF for almost all variables increased over 1, 3, and 5 years, and was higher for larger tumors and in black patients, unmarried patients, and patients who had not undergone surgery, had received radiation, and had had their regional lymph nodes examined. The CIF values in AJCC stages I, II, III, IV were 2.5%, 7.0%, 12.1%, and 34.2% respectively (*P* < .001). There was also a significant difference in the degree of lymphatic metastasis and a CIF of death from penile cancer (*P* < .001). The CIF was 56.2% for a follow‐up time of 5 years for distant metastasis. The data are listed in detail in Table 2.

**Table 2:** Univariable analysis in patients with penile cancer by using competing risk model

Variables	Gray's test	*P*‐value	Cumulative incidence function		
			12‐mo	36‐mo	60‐mo
Age	116.37	**<.05**	…	…	…
Race	6.46	**.04**			
White			0.08	0.16	0.19
Black			0.12	0.23	0.25
Other			0.06	0.13	0.14
Marital status	1.07	.59			
Married			0.09	0.17	0.20
Unmarried			0.10	0.19	0.20
DSW			0.08	0.17	0.20
AJCC Stage	206.07	**<.001**			
I			0.03	0.07	0.09
II			0.07	0.16	0.20
III			0.12	0.24	0.27
IV			0.34	0.45	0.45
Surgery	4.98	**.03**			
Yes			0.08	0.16	0.19
No/Unknown			0.16	0.25	0.27
Radiation	28.08	**<.001**			
Yes			0.17	0.32	0.34
No/Unknown			0.08	0.15	0.17
Tumor size(cm)	63.20	**<.001**			
≤1			0.03	0.08	0.09
1 < T≤ 3			0.05	0.11	0.14
3 < T≤5			0.10	0.28	0.30
>5			0.19	0.28	0.30
N	251.70	**<.001**			
N0			0.05	0.07	0.13
N1			0.11	0.201	0.37
N2			0.24	0.35	0.45
N3			0.40	0.49	0.53
M	89.92	**<.001**			
M0			0.07	0.15	0.18
M1			0.49	0.56	0.56
Regional nodes examined	45.36	**<.001**			
Yes			0.12	0.25	0.29
No			0.07	0.13	0.15
Primary site	1.17	.76			
Glans penis			0.08	0.16	019
Body of penis			0.07	0.16	0.19
Overlapping lesion of penis			0.09	0.18	0.23
Penis NOS			0.09	0.16	0.19

Abbreviations: AJCC, American Joint Committee on Cancer; DSW, divorced & separated &widowed; NOS, not otherwise specified.

Bold values means that *P* < .05.

### 3.3 Multivariate analysis of the prognosis of penile cancer

When competing events were present, we included variables that were statistically significant in the univariate analysis in the Fine‐Gray model. The multivariate analysis indicated that being black, AJCC stage II, AJCC stage III, tumor size > 5 cm, and TNM stages N1, N2, N3, and M1 were significantly associated with survival. The data are listed in detail in Table 3, which includes the results from the multivariate Cox regression for comparison. Due to age as a prognostic factor changing with time and causing variations in other prognostic factors, we repeated the Fine‐Gray analysis using age as a time‐varying covariate. Variables in multiple categories in that analysis were converted into two‐category dummy variables. In the presence of competing risks, a stepwise regression method is used for screening based on Bayesian information criteria. The results showed that when age was used as a time covariate, AJCC stage II (vs AJCC stage I: hazard ratio [HR] = 1.85, 95% confidence interval [CI] = 1.30‐2.63, *P* < .001), AJCC stage III (vs AJCC stage I: HR = 1.88, 95% CI = 1.29‐2.74, *P* < .001), tumor size > 5 cm (vs tumor size ≤ 1 cm: HR = 2.35, 95% CI = 1.40‐3.94, *P* < .05), TNM stage N1 (vs TNM stage N0: HR = 2.24, 95% CI = 1.58‐3.19, *P* < .001), TNM stage N2 (vs TNM stage N0: HR = 2.96, 95% CI = 2.04‐4.28, *P* < .001), TNM stage N3 (vs TNM stage N0: HR = 4.60, 95% CI = 2.50‐8.45, *P* < .001), and TNM stage M1 (vs TNM stage M0: HR = 2.13, 95% CI = 1.23‐3.70, *P* < .05) exerted statistically significant effects in penile cancer. Significant differences were also found in the stratification of each prognostic factor (Figure [Link], [Link], [Link], [Link]). Neither the linear term (relative risk [RR] = 1.00, *P* = .73) nor the quadratic term (RR = 1.00, *P* = .62) expressing the interaction of time with age was statistically significant. The data are listed in detail in Table 4.

**Table 3:** Multivariable analysis in patients with penile cancer

Variables	Fine‐Gray regression analysis			COX regression analysis		
	HR	(95% CI)	*P*‐value	HR	(95% CI)	*P*‐value
Age	1.00	0.99‐1.00	.44	1.04	1.03‐1.05	**<.001**
Race						
White	Reference			Reference		
Black	1.51	1.10‐2.07	**.01**	1.38	1.11‐1.71	**<.05**
Other	0.74	0.45‐1.22	.23	1.05	0.79‐1.38	.77
Marital status						
Married	…			Reference		
Unmarried				1.16	0.96‐1.42	.13
DSW				1.19	1.01‐1.34	**.03**
AJCC Stage						
I	Reference			Reference		
II	1.94	1.36‐2.77	**<.001**	1.15	1.00‐1.44	.17
III	1.98	1.34‐2.91	**<.001**	1.33	1.07‐1.64	.010
IV	1.64	0.87‐3.12	.13	1.45	0.95‐2.21	.08
Surgery						
Yes	0.86	0.52‐1.44	.57	0.63	0.46‐0.86	**<.05**
No/Unknown	Reference			Reference		
Radiation						
Yes	1.13	0.82‐1.58	.45	0.98	0.78‐1.23	.83
No/Unknown	Reference			Reference		
Tumor size(cm)						
≤1	Reference			Reference		
1 < T≤ 3	1.24	0.76‐2.04	.39	1.33	1.02‐1.72	**.03**
3 < T≤5	1.55	0.93‐2.57	.09	1.62	1.23‐2.12	**<.05**
>5	2.23	1.33‐3.72	**<.05**	2.00	1.50‐2.67	**<.001**
N						
N0	Reference			Reference		
N1	2.49	1.71‐3.61	**<.001**	1.71	1.33‐2.200	**<.001**
N2	3.25	2.18‐4.84	**<.001**	1.73	1.32‐2.26	**<.001**
N3	5.05	2.69‐9.50	**<.001**	2.72	1.48‐3.22	**<.001**
M						
M0	Reference			Reference		
M1	2.21	1.28‐3.84	**<.05**	2.18	1.48‐3.22	**<.001**
Regional nodes examined					…	
Yes	0.81	0.61‐1.07	.14			
No	Reference					

Abbreviations: 95% CI, 95% confidence interval; AJCC, American Joint Committee on Cancer; DSW, divorced & separated &widowed; HR, hazard ratio; NOS, not otherwise specified. Bold values means that *P* < .05.

**Table 4:** Multivariable analysis by Bayesian Information Criterions for competing risk

Variables	HR	(95% CI)	*P*‐value
*Age.t	1.00	1.00‐1.00	.73
*Age.t^2^	1.00	1.00‐1.00	.62
AJCC stage			
I	Reference		
II	1.85	1.30‐2.63	**<.001**
III	1.88	1.29‐2.74	**<.001**
IV	1.67	0.88‐3.18	.12
Tumor size(cm)			
≤1	Reference		
1 < T ≤ 3	1.30	0.79‐2.14	.30
3 < T ≤ 5	1.64	0.99‐2.73	.06
>5	2.35	1.40‐3.94	**<.05**
N			
N0	Reference		
N1	2.24	1.58‐3.19	**<.001**
N2	2.96	2.04‐4.28	**<.001**
N3	4.60	2.50‐8.45	**<.001**
M			
M0	Reference		
M1	2.13	1.23‐3.70	**<.05**

Abbreviations: 95% CI, 95% confidence interval; AJCC, American Joint Committee on Cancer; HR, hazard ratio.

* Age was used as time‐varying covariates.

Bold values means that *P* < .05

### 3.4 Comparative analysis

**Figure 2:**
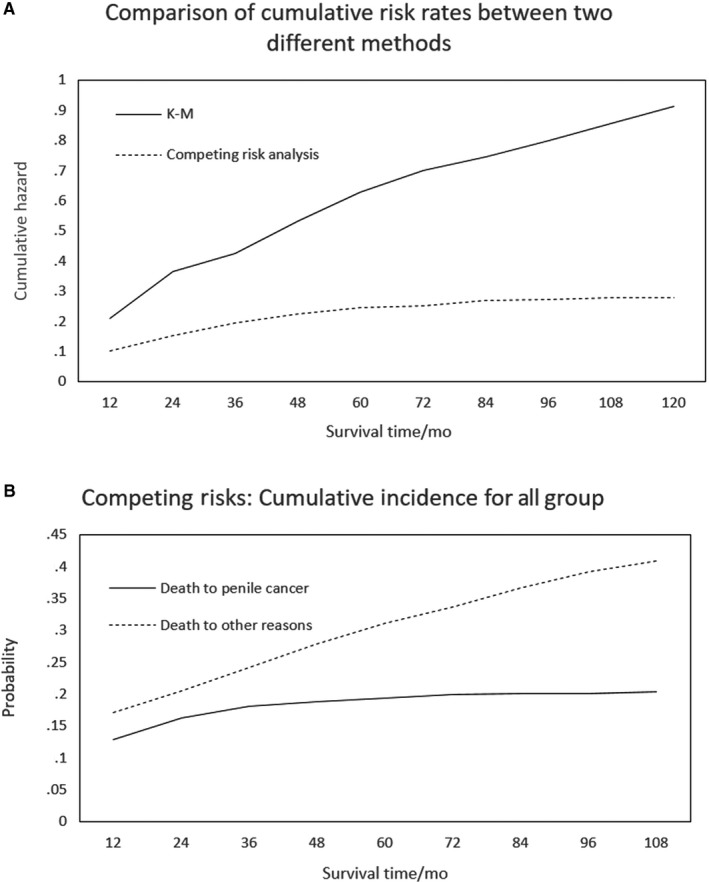
Comparative analysis

We compared the results from classical Kaplan‐Meier curve analysis with the cumulative risk rate of the competing‐risks model, which revealed that only Kaplan‐Meier curve analysis led to an overestimation of the cumulative risk of the patient (Figure 2A). The results show that, in fact, when there is a risk of competition, the cumulative risk of penile cancer patients is not as high as the cumulative risk of the K‐M method. As can be seen from Figure 2B, the cumulative incidence due to death from other causes for the same survival time was higher than that from penile cancer alone. If death from other causes is treated as censored, it will have a greater impact on the results.

## 4 DISCUSSION

Single endpoints are rarely observed in medical research, with instead multiple endpoints that compete with each other commonly being present. The occurrence of competing events hinders analyses of the occurrence of ending events of interest in a study.^21^, ^22^ Previous studies have widely used Kaplan‐Meier estimates of survival curves and Cox regression models to describe survival trends and identify important prognostic factors.^21^ In the real world, the research object not only experiences one type of event, but different types of ending events affect each other, that is, form competing events. The statistical model for processing data with competitive events is called the “competing risk model.” Survival data positive events usually include all‐cause death and cause‐specific death. When the study does not involve competing risks, K‐M, COX regression method can be used for research. However, medical research generally has competing risks. When discussing specific causes of death, the traditional method may overestimate the cumulative incidence of each variable. It is therefore necessary to use the competing‐risks model to deal with multiple end events.^23^, ^24^ In our study, competing risk analysis did not consider events due to penile cancer death. It also considers events that die for other reasons and the effects of events.

Penile cancer is a relatively rare malignant tumor in the United States, and a delayed or missed diagnosis can lead to post treatment dysfunction and reduced survival.^25^, ^26^ Our study included 874 penile cancer patients who had died between 2004 and 2015, with 541 dying of other causes such as other cancers, suicides, and accidents, while 333 had died of their penile cancer. This means that less than two‐thirds of the results of interest, indicating the need to apply a competing‐risks model. Two main methods are currently used to analyze competing risks: the cause risk model and the cumulative risk model. The cumulative risk model considers other competing endpoint events simultaneously when calculating an endpoint event of interest, which is more realistic**.** The present study is the first to conduct a risk analysis of penile cancer patients using a cumulative risk model in a competing‐risks model and thereby identifying more accurate prognostic factors.

Sharma et al found that being black was associated with worse overall survival among penile cancer patients (HR = 1.39, 95% CI = 1.21‐1.58, *P* < .01),^27^ while Slopnick et al found that that African‐American penile cancer patients had higher mortality rates than white patients (HR = 1.25, 95% CI = 1.10‐1.42, *P* < .001).^28^ These results are based only on Cox regression analysis and are similar to our Cox regression results (HR = 1.38, 95% CI = 1.11‐1.72, *P* < .05), and our Fine‐Gray regression analysis revealed that the Cox regression model underestimated the risk of black race (HR = 1.51, 95% CI = 1.10‐2.07, *P* < .05).

The Cox regression model also underestimates the risk of the AJCC stage. The Fine‐Gray model revealed that AJCC stage II (HR = 1.94, 95% CI = 1.36‐2.77, *P* < .001) and AJCC stage III (HR = 1.98, 95% CI = 1.34‐2.91, *P* < .001) were risk factors for death in penile cancer patients compared with AJCC stage I. However, in the Cox regression, we did not observe statistical significance for AJCC stage II, and the HR for AJCC stage III was only 1.33. The HR for AJCC stage IV was >1 in both the Fine‐Gray model and the Cox regression model, although we did not find that AJCC stage IV was statistically significant. We believe that these findings may have resulted from the interaction of multiple variables included in the model, and so these relationships need to be explored further.

The Fine‐Gray model indicated that the *p* value was statistically significant only for a tumor size of >5 cm. The N stage is a very significant prognostic factor, with lymph node metastasis long having been considered an important prognostic factor for penile cancer.^29^, ^30^ TNM stages N0, N1, N2, and N3 all significantly affect survival in the present study, whereas Cox regression underestimated the risk of the N stage. Finally, we also found that distant metastasis is a risk factor affecting patient survival (HR = 2.22, 95% CI = 1.28‐3.84, *P* < .05). These observations indicate that the relative risk of a patient dying from penile cancer when a competing event is present is different from when considering only a single endpoint event.

Our univariate analysis indicated that age was a statistically significant factor. Since age increases over time and so may result in changes in other prognostic factors, we performed a multivariate analysis of age as a time‐varying covariate. The results showed that AJCC stage II, AJCC stage III, tumor size > 5 cm, and TNM stages N1, N2, N3, and M1 were significantly associated with survival. Age was not an independent prognostic factor affecting survival and its effect did not change over time. This is the first study to use the competing‐risks model to analyze the survival of patients with penile cancer. While we conclude from the present results that age is not a prognostic factor for patients, this relationship needs to be studied further.

When a competing event exists, the incidence of events of interest at time *t* in the cumulative risk model is conditional on the composite event rate of all events of interest and those competing events, whereas the Kaplan‐Meier estimation is only conditional on the incidence of events of interest. We compared the results from classical Kaplan‐Meier curve analysis with the cumulative risk rate of the competing‐risks model. When a competing event is treated as censored data, using the Kaplan‐Meier method to calculate the cumulative risk results in a larger effect than the cumulative risk calculated using the competing‐risks model, thereby overestimating the actual situation.

One major strength of the present study was that the SEER database provides a very large number of samples to explore risk factors and construct accurate competing‐risks models. However, it is undeniable that our research was subject to some limitations. First, there are no records in the SEER database for certain common variables related to a prognosis, such as chemotherapy status, smoking history, and vaccine status. Second, the data used in this study were for patients diagnosed with penile cancer between 2004 and 2015, and so the relatively short follow‐up period might have also affected the estimation of cumulative incidence. Finally, because this study is the first to use a competing‐risks model for risk assessment of penile cancer patients, further research is needed to validate its findings.

In conclusion, this study established a competing‐risks analysis model for the first time based on the SEER database for risk assessments of penile cancer patients. The obtained results may help clinicians to better understand penile cancer and provide these patients with more appropriate support.

## CONFLICT OF INTEREST

The author reports no conflicts of interest in this work.
