# Supplementary figures and images for: Competing‐risks model for predicting the prognosis of penile cancer based on the SEER database
Source: Cancer Med. 2019 Oct 27;8(18):7881–9. doi: 10.1002/cam4.2649 (PMC6912058; doi:10.1002/cam4.2649)

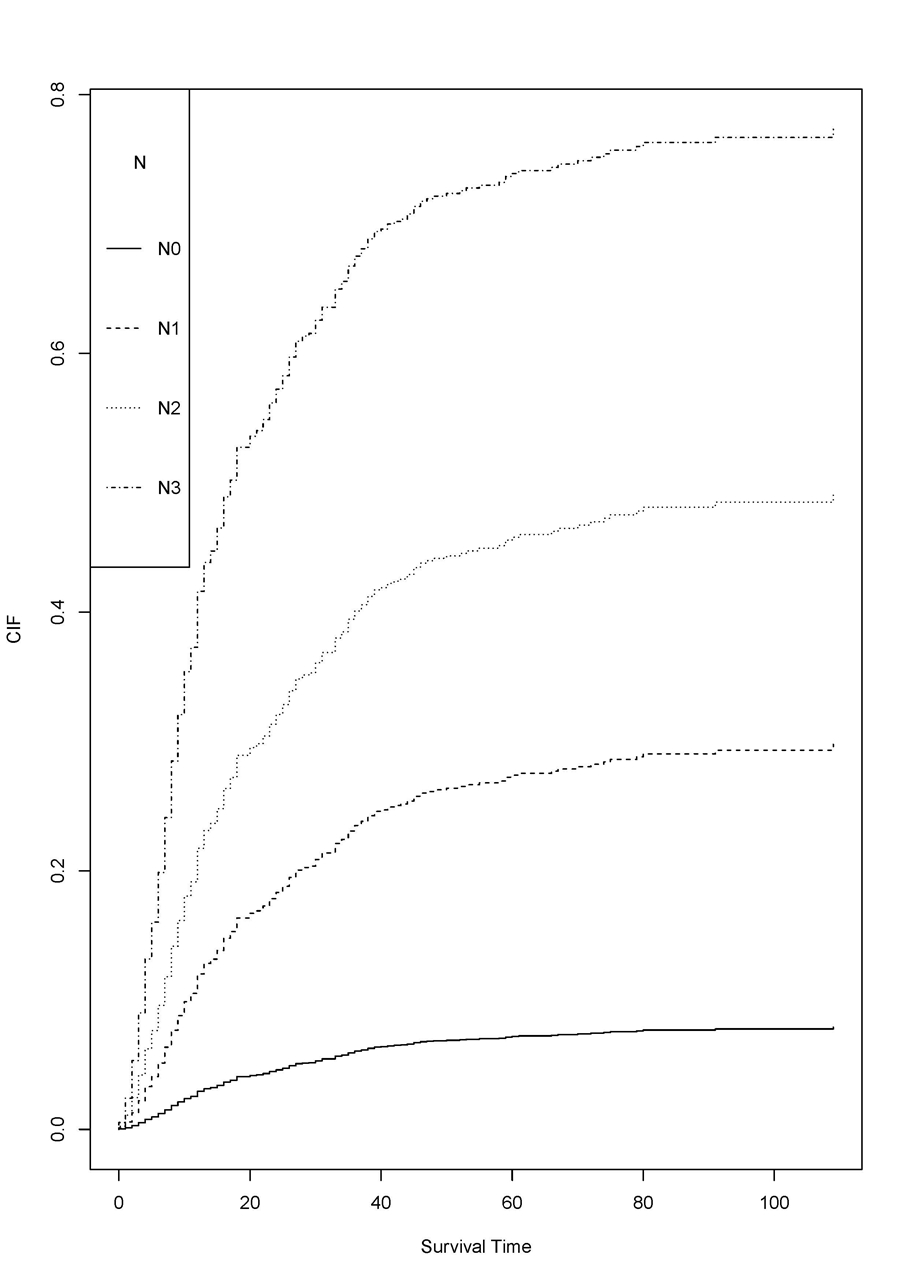

Supplement: Supplementary file 1 [file CAM4-8-7881-s001.tif]

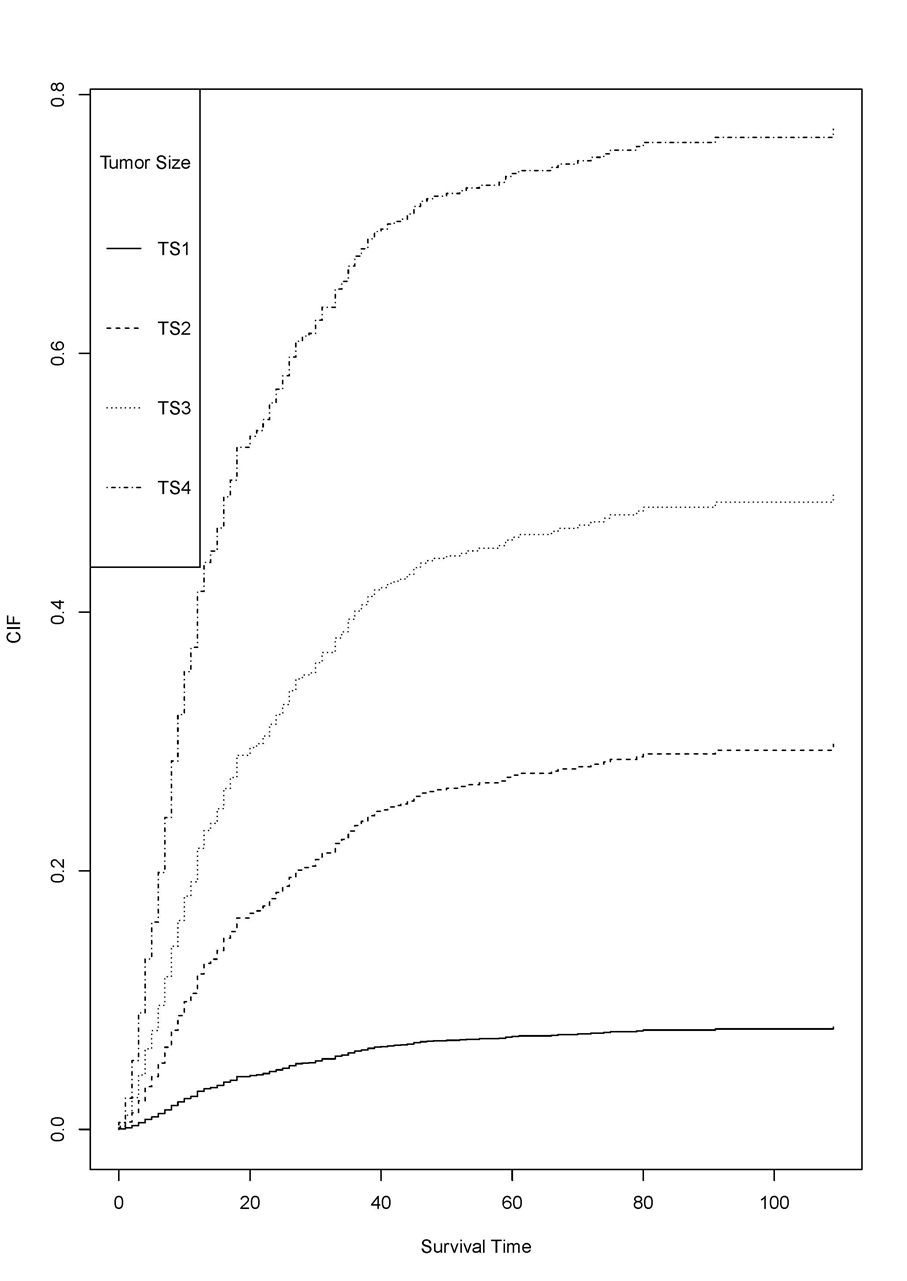

Supplement: Supplementary file 2 [file CAM4-8-7881-s002.tif]

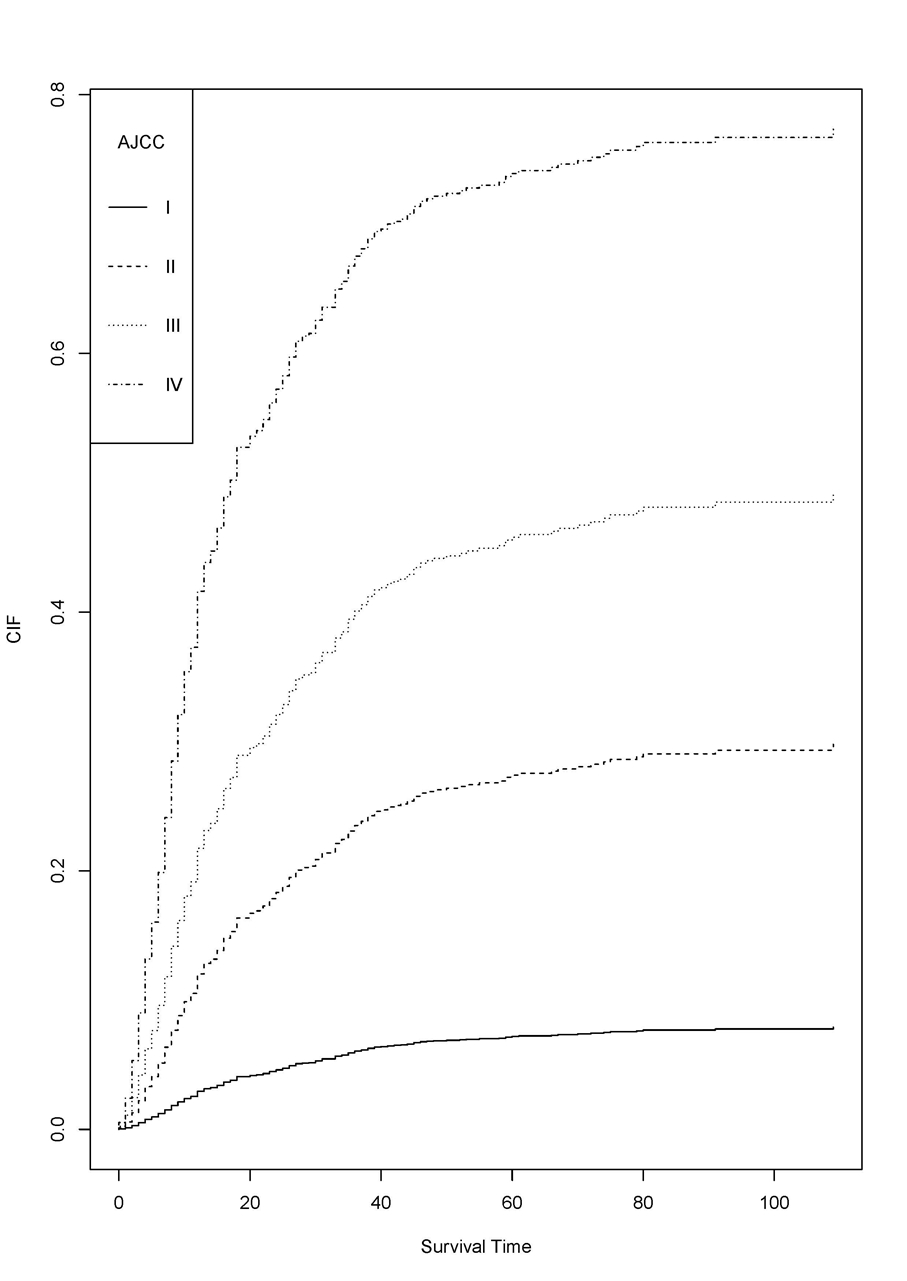

Supplement: Supplementary file 3 [file CAM4-8-7881-s003.tif]

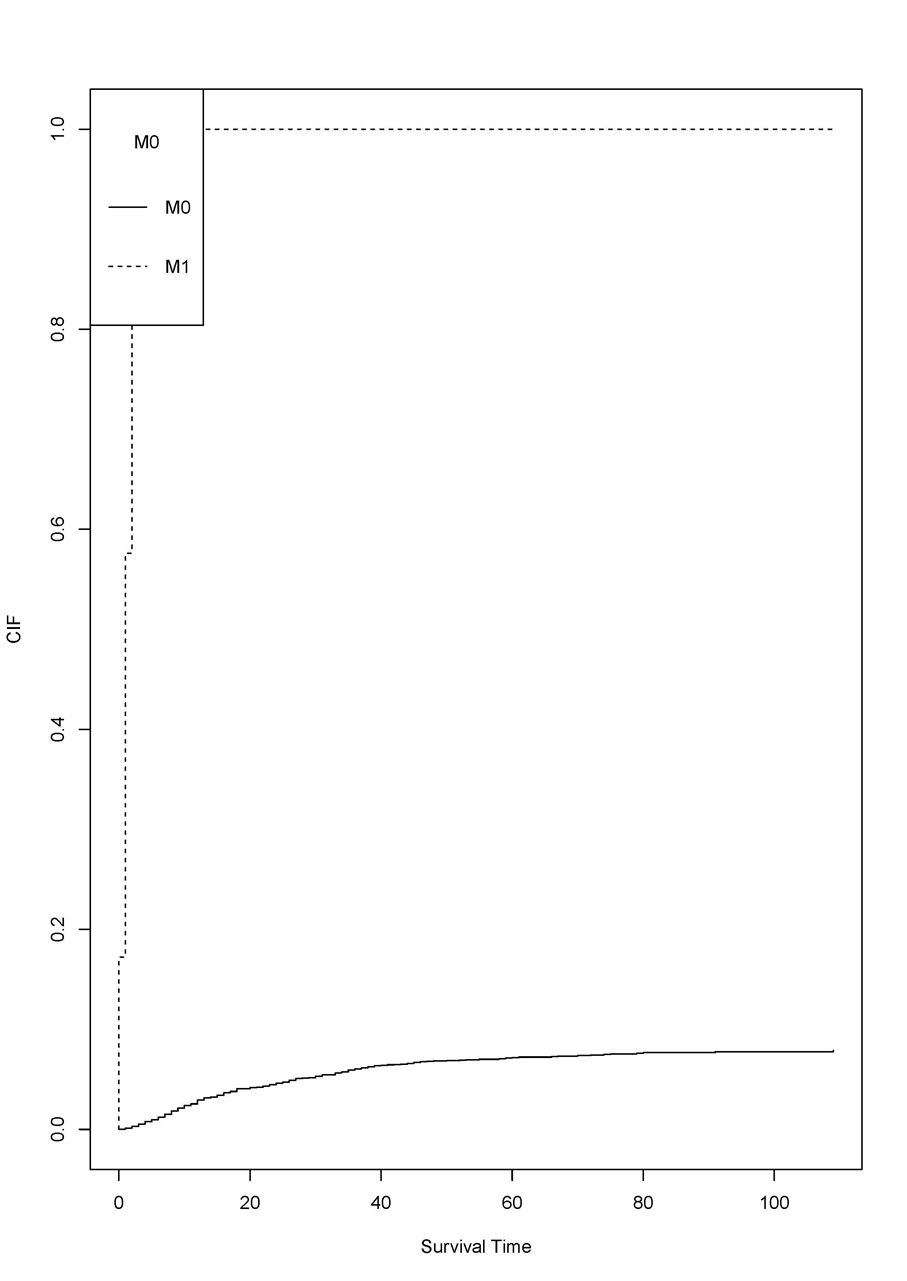

Supplement: Supplementary file 4 [file CAM4-8-7881-s004.tif]
